# Online IMU Self-Calibration for Visual-Inertial Systems

**DOI:** 10.3390/s19071624

**Published:** 2019-04-04

**Authors:** Yao Xiao, Xiaogang Ruan, Jie Chai, Xiaoping Zhang, Xiaoqing Zhu

**Affiliations:** 1 Faculty of Information Technology, Beijing University of Technology, Beijing 100124, China; xiaoyao1103@emails.bjut.edu.cn (Y.X.); adrxg@bjut.edu.cn (X.R.); chaijie@emails.bjut.edu.cn (J.C.); 2 College of Electrical and Control Engineering, North China University of Technology, Beijing 100144, China; zhangxiaoping369@163.com

**Keywords:** visual-inertial system, visual odometry, SLAM, IMU calibration, sensor fusion

## Abstract

Low-cost microelectro mechanical systems (MEMS)-based inertial measurement unit (IMU) measurements are usually affected by inaccurate scale factors, axis misalignments, and g-sensitivity errors. These errors may significantly influence the performance of visual-inertial methods. In this paper, we propose an online IMU self-calibration method for visual-inertial systems equipped with a low-cost inertial sensor. The goal of our method is to concurrently perform 3D pose estimation and online IMU calibration based on optimization methods in unknown environments without any external equipment. To achieve this goal, we firstly develop a novel preintegration method that can handle the IMU intrinsic parameters error propagation. Then, we frame IMU calibration problem into general factors so that we can easily integrate the factors into the current graph-based visual-inertial frameworks and jointly optimize the IMU intrinsic parameters as well as the system states in a big bundle. We evaluate the proposed method with a publicly available dataset. Experimental results verify that the proposed approach is able to accurately calibrate all the considered parameters in real time, leading to significant improvement of estimation precision of visual-inertial system (VINS) compared with the estimation results with offline precalibrated IMU measurements.

## 1. Introduction

Vision-based motion estimation and 3D reconstruction is a very active field of research in robotics and computer vision communities over the last decades due to its potential applications, such as robot navigation, autonomous driving, augmented reality (AR) and virtual reality (VR). Different sensor setups can be used for vision-based motion estimation: monocular [1,2], stereo [3,4], RGB-D [5,6] and visual-inertial [7,8]. Among these, the combination of cameras and inertial sensors, also known as visual-inertial system (VINS), has attracted more and more attention in recent years [7,8,9,10,11,12,13,14,15,16,17,18,19,20,21]. On the one hand, camera and inertial measurements offer complementary nature, and the combination of these two sensors can lead to significant improvements in accuracy of the estimation results and robustness of the system. On the other hand, with the development of microelectro mechanical systems (MEMS) inertial sensors, the cost of inertial measurement unit (IMU) steadily decline and an increasing number of robot platforms, drones and consumer electronics, especially mobile devices, are equipped with low-cost MEMS-based IMUs.

Due to the imperfections of the manufacturing and physical characteristics of the sensors, real IMU measurements are usually affected by systematic errors as well as random noise [22,23]. The process of identifying the intrinsic parameters of sensors for compensating these errors is known as IMU calibration. Although VINS have seen tremendous improvements in accuracy, robustness and efficiency over recent years, most visual-inertial methods either treat these measurement errors as sensor noise or assume the IMU that used is precalibrated offline. For high performance inertial sensors, which generally are manufactured precisely or factory calibrated carefully with each sensor is sold with its own calibration parameters stored into the firmware, providing accurate measurements off the shelf [23], this assumption is reasonable. However, for low-cost MEMS-based inertial sensors, which are usually used for consumer mobile robots, neglecting these errors may significantly influence the performance of VINS or even breakdown the whole system. Moreover, offline IMU calibration have certain shortcomings. Traditional high precision calibration process heavily rely on special external equipment such as robot manipulator [24,25,26], high accuracy turntable [27], motion tracked system [28]. Such equipment is usually very expensive. As a result, the cost of one calibration process often exceeds the cost of MEMS-based IMU. Secondly, the IMU measurement models used for calibration are usually simplified linear models, the intrinsic parameters of which may vary with mechanical shocks, temperature and other factors. Treating these parameters as constant would lead to unmodeled errors and degenerate the performance of the VINS. Therefore, even high quality offline-calibration is performed, this process still needs to be repeated periodically. Moreover, performing offline calibration often needs special operators and is time consuming, which hinders the rapid deployment of VINS to consumer devices.

To this end, we propose an online IMU self-calibration method for VINS. Specifically, we are interested in concurrently performing 3D pose estimation and online IMU calibration in unknown environment, using only camera and inertial measurements, and without any knowledge about the structure of the scene or any external equipment.

We implement the method with the extension of an open source monocular VINS pipeline, VINS-Mono [8], and demonstrate that, in this setting, it is possible to concurrently localize and perform online calibration of all of the following quantities: the axis misalignments and scale factors of the IMU sensors, the g-sensitivity (also called linear acceleration dependence) of the gyroscopes. What we have to claim is that although our implementation is based on VINS-Mono, the proposed online IMU self-calibration method can be easily integrated into other graph-based visual/visual-inertial frameworks as well as the multi-camera cases. To the best of our knowledge, this is the first paper to present a VINS pipeline with online IMU self-calibration based on optimization methods. We highlight our main contributions as follows:

1. An online IMU self-calibration method for visual-inertial system that can perform in real time in unknown environment without any external equipment.
2. We frame the IMU calibration problem into general factors so that the proposed method can be easily integrated into other graph-based visual/visual-inertial frameworks.
3. An extensive evaluation on publicly available dataset showing that the online calibration can obtain accurate IMU intrinsics estimation and significantly improve the estimation precision of the VINS method compared with the estimation results with offline precalibrated IMU measurements.

The rest of this paper is organized as follows. In Section 2, we discuss the relevant literature. The algorithm as well as the implementation details are introduced in Section 3. The experimental results are shown in Section 4. Finally, the paper is concluded with the discussion and possible future research directions in Section 5.

## 2. Related Work

Over the last decade, there has been tremendous progress in VINS. Existing approaches can be classified into filtering-based methods [9,10,11,12,13,14,15,16] and graph-based methods [7,8,17,18,19,20,21]. Filtering-based methods usually process the fusion with the extended Kalman filter (EKF) and its variants, in which, the measurements from IMU are used for state propagation and observations from vision are used for update. Filtering-based methods have the advantage of fast processing since it contentiously marginalizes past states. However, their performance are usually sub-optimization due to early fix of linearization points. Compared to filter-based methods, graph-based methods have attracted more attention in the recent years, as they obtain more accurate estimation results by maintaining measurements of long term history and perform batch optimization to obtain the optimal estimate. To achieve real time operation, graph-based methods usually apply keyframe-based techniques and optimize over a bounded-size sliding window of recent states by marginalizing past states and measurements. Nevertheless, all of these methods above either treat the IMU measurement errors as sensor noise or assume the IMU that used is precalibrated offline except the work in [13], which, to the best of our knowledge, is the only VINS method that implement IMU and camera online self-calibration in a filter-based framework. However, only simulation results were shown in this paper. Besides, their method cannot be applied for graph-based VINS methods.

For offline IMU calibration, numerous methods have been proposed over the last several decades, with the development of strapdown inertial navigation system or AHRS [22]. As we are concerned with online self-calibration, here we only give a brief review of these methods. Traditionally IMU calibration methods are usually done by using special external equipment that could provide known reference acceleration or rotational velocity of the inertial sensor. The measurements of the sensor are compared directly with the known reference value to determine the intrinsic parameters. Apparently, the accuracy of these methods strongly relies on the accuracy of the kinematic reference. To obtain highly accurate results, expensive mechanical platforms, such as robot manipulator or high accuracy turntable [24,25,26,27] are usually used, resulting in a calibration cost that often exceeds the cost of the IMU‘s hardware. In [28] the authors exploited using a marker-based optical tracking system to provide the reference value, while in [29], the GPS readings are used to calibrate initial biases and misalignments. In order to achieve in-situ calibration, the multi-position method was firstly introduced in [30], using the fact that the magnitude of the static acceleration must equal to the gravity‘s magnitude. However, this method can only calibrate the bias and scale factors of the accelerometer. This technique has been further extended by [31], in which, a method with two calibration schemes was presented. The accelerometer is firstly calibrated by exploiting the high local stability of the gravity vector‘s magnitude, and then the gyroscope is calibrated by comparing the gravity vector sensed by the calibrated accelerometer with the gravity vector obtained by integrating the angular velocities. The main advantage of this method is that it do not require any external mechanical equipment. A similarly approach also can be found in [23]. A self-calibration method based on an iterative matrix factorization was proposed by Hwangbo et al. [32]. this method use gravity as accelerometers reference, and a camera as gyroscopes reference. A noteworthy work in the VINS domain is the kalibr toolbox by Rehder et al. [33], in which, multiple camera-IMU extrinsic as well as the IMU intrinsics are jointly optimized in a single estimator with the continues-time batch optimization framework [33,34]. Impressive results were demonstrated in this work. However, their method cannot be integrated directly in the presented discrete-time graph-based VINS methods.

Motivated by IMU preintegration theories developed by [8,35,36,37,38], we firstly develop a novel preintegration method to handle the IMU intrinsic parameters error propagation. Then we frame the IMU preintegration measurements and IMU intrinsic parameters into general factors. In such a way, we can implement an optimization method to jointly estimate the IMU intrinsic parameters as well as the system state in a big bundle, which makes our method totally distinguished from the method in [13]. Compared to the the offline IMU calibration methods, our method integrates into the current VINS method and performs online IMU self-calibration, without any special equipment.

## 3. Methodology

### 3.1. Visual-Inertial System

**Figure 1:**
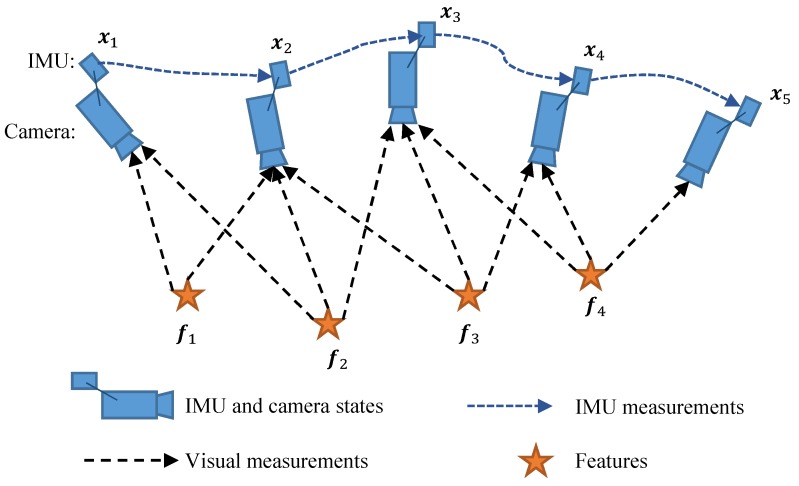
Illustration of visual-inertial system. Several keyframes and inertial measurement unit (IMU) measurements are maintained in a sliding window. When a new keyframe is inserted into the sliding window, a local bundle adjustment (BA) would be implemented to jointly optimize the camera and IMU states, as well as the feature location.

Consider a sensing system (e.g., a mobile robot or a hand-held device) equipped with a monocular camera and a low-cost uncalibrated IMU. Our goal is to concurrently estimate the states of the sensing system, as well as the IMU intrinsics in a tightly-coupled graph-based framework. A general visual-inertial system is illustrated in Figure 1, in which, several keyframes and IMU measurements are maintained in a sliding window. When a new keyframe is inserted into the sliding window, a local bundle adjustment (BA) would be implemented to jointly optimize the camera and IMU states, as well as the feature location. After optimization, one of the old keyframe as well as its corresponding states and measurements would be marginalized from the sliding windows, limiting the sliding windows size and reducing computation complexity.

The notations and frame definitions used in this paper are briefly described as follows. We denote the world frame and the body frame (BF) as ${\left(\cdot \right)}^{w}$ and ${\left(\cdot \right)}^{b}$, respectively. The z-axis of the world frame is aligned with the direction of the gravity. The BF is defined to be the same as the accelerometer orthogonal frame (AOF). We primarily use quaternions q with Hamilton convention to represent rotation, but rotation matrices R are also used for the convenient representation of 3D vectors rotation. $q_{b}^{w}$ represents the orientation of body frame with respect to the word frame. $R_{b}^{w}$ is the corresponding rotation matrix of $q_{b}^{w}$. The parameter $p_{b}^{w}$ is the translation from the world frame to the body frame, being expressed in word frame, $b_{k}$ is the body frame while taking the *k*-th image, $g^{w}$ and $g^{b}$ are the gravity vectors in the world frame and body frame, respectively.

The full state vector in the sliding window is defined as

(1) $$\begin{array}{cc} X & =[x_{0},x_{1},\cdots ,x_{n},x_{I},f_{0},f_{1},\cdots ,f_{m}]\\ x_{k} & =[p_{b_{k}}^{w},q_{b_{k}}^{w},v_{b_{k}}^{w},b_{a},b_{\omega }], \end{array}$$

where $x_{k}$ is the IMU state at the *k*-th image. It contains position, orientation and velocity of the IMU as well as the accelerometer bias $b_{a}$ and gyroscope bias $b_{\omega }$. The parameter $x_{I}$ is the IMU intrinsic parameters, $f_{l}$ is the feature location, which can be parameterized by either the 3D position or inverse depth of the feature, *n* is the number of keyframes in the sliding window, *m* is the number of features which have been observed at least by two frames in the sliding window.

After the VINS was successfully initialized with the method proposed in [8,39], we were able to use a sliding window based framework to jointly optimize both the full system states as described in Equation (1). We minimize the sum of prior and the Mahalanobis norm of all measurement residuals to obtain a maximum posteriori estimation as

(2) $$\min_{X}\left\{{∥r_{p}-H_{p}X∥}^{2}+\sum_{k\in B}{∥r_{B}\left({\hat{z}}_{b_{k+1}}^{b_{k}},X\right)∥}_{P_{b_{k+1}}^{b_{k}}}^{2}+\sum_{(l,j)\in C}{∥r_{C}\left({\hat{z}}_{l}^{c_{j}},X\right)∥}_{P_{l}^{c_{j}}}^{2}+{∥r_{I}\left(x\right)∥}_{P_{I}}^{2}\right\},$$

where $\left\{r_{p},H_{p}\right\}$ are the prior information from marginalization. $r_{B}\left({\hat{z}}_{b_{k+1}}^{b_{k}}X\right)$ and $r_{C}\left({\hat{z}}_{l}^{c_{j}},X\right)$ are residuals for the IMU and visual measurements respectively, where B is the set of all IMU measurements and C is the set of features. The parameter $r_{I}\left(x\right)$ is the residuals for IMU intrinsic parameters.

As our goal is to estimate the IMU intrinsic parameters online, in this paper, we assume the translation and orientation between camera and IMU are fixed and known from prior calibration. Furthermore, we assume there is a vision front-end for feature detection and tracking, providing the pixel measurements of the features. Therefore, we will neglect the residuals of vision measurements $r_{C}\left({\hat{z}}_{l}^{c_{j}},X\right)$ in this paper and only detail the residuals of IMU measurements $r_{B}\left({\hat{z}}_{b_{k+1}}^{b_{k}}X\right)$ as well as the IMU intrinsics $r_{I}\left(x\right)$ in the following.

### 3.2. IMU Model

A six-degree-of-freedom IMU composes by a triple axis accelerometer and a triple axis gyroscope. Ideally, the two triple-axis define a single, shared, orthogonal 3D frame. The accelerometer senses the acceleration of the sensor along of each input axis, while gyroscope measures the angular velocity around each input axis. The scale factors convert analog or digital output of the sensor to real physical quantity measurements. Unfortunately, due to assembly imperfections, each axis of the accelerometer and gyroscope deviates by a small angle from its designed mounting direction, as a result, introducing axis misalignment and cross-axis sensitivity errors to the measurements. Also, real scale factor of each axis is unique and different from the nominal value provided by the manufacturer. In additional, the angular velocities measurements are affected by the accelerations that the sensor is subjected. Moreover, all of the measurements are almost always affected by variable bias. In order to model and compensate these errors, we employ the IMU measurement model as described in [33].

(3) $$\begin{array}{cc} a_{m} & =S_{a}M_{a}\left(a_{wb}^{b}-g^{b}\right)+b_{a}+n_{a} \end{array}$$

(4) $$\begin{array}{cc} \omega_{m} & =S_{\omega }M_{\omega }\omega_{wb}^{b}+A_{\omega }\left(a_{wb}^{b}-g^{b}\right)+b_{\omega }+n_{\omega }, \end{array}$$

where $a_{m}$ and $\omega_{m}$ are the measurement of accelerometer and gyroscope, respectively, $S_{a}$ is a 3×3 diagonal matrix constructed by the scale factors of the triple axis, $M_{a}$ is a 3×3 lower triangular matrix representing the axis misalignments and the cross-coupling terms, and $S_{\omega }$ and $M_{\omega }$ are defined analogously to $S_{a}$ and $M_{a}$. Each row of $M_{a}$ and $M_{\omega }$ is a unit vector. The parameter $A_{\omega }$ is a full populated matrix representing the g-sensitivity coefficients of gyroscope, $a_{wb}^{b}$ and $\omega_{wb}^{b}$ are the body frame acceleration and angular velocity with respect to the word frame, being expressed in the body frame, $b_{a}$ and $b_{\omega }$ is the measurement bias of accelerometer and gyroscope respectively, $n_{a}$, $n_{\omega }$ is the measurement noise. We assume that $n_{a}$, $n_{\omega }$ are Gaussian white noise, $n_{a}∼N\left(0,\sigma_{a}^{2}\right)$, $n_{\omega }∼N\left(0,\sigma_{\omega }^{2}\right)$. For the derivations of Equations (3) and (4), please refer to [33].

The accelerometer and gyroscope bias are modeled as random walks, the derivatives of which are Gaussian white noise, $n_{b_{a}}∼N\left(0,\sigma_{b_{a}}^{2}\right)$, $n_{b_{\omega }}∼N\left(0,\sigma_{b_{\omega }}^{2}\right)$.

(5) $${\dot{b}}_{a}=n_{b_{a}},\;{\dot{b}}_{\omega }=n_{b_{\omega }}.$$

It is inconvenient to optimize the $M_{a}$ and $M_{\omega }$ as each row of these two matrices is constrained to a unit vector. Besides, when integrating the angular velocity and acceleration to obtain the motion of the system, we need to compute the inverse matrix of $S_{a}$, $M_{a}$, $S_{\omega }$, $M_{\omega }$ at every step to compensate the measurement errors, which is a waste of computing resources. If defining $T_{a}={\left(S_{a}M_{a}\right)}^{-1}$, $T_{\omega }={\left(S_{\omega }M_{\omega }\right)}^{-1}$, we could get the compensated acceleration and angular velocity from the measurements more effectively:

(6) $$\begin{array}{cc} a_{wb}^{b} & =T_{a}\left(a_{m}-b_{a}-n_{a}\right)+g^{b} \end{array}$$

(7) $$\begin{array}{cc} \omega_{wb}^{b} & =T_{\omega }\left(\omega_{m}-A_{\omega }\left(a_{wb}^{b}-g^{b}\right)-b_{\omega }-n_{\omega }\right), \end{array}$$

where, $T_{a}$ is still a lower triangular matrix, $T_{\omega }$ is a full populated matrix without constraint. For an uncalibrated IMU, $T_{a}$, $T_{\omega }$ is roughly close to identify matrix with some uncertainty. $A_{\omega }$ is close to 0.

### 3.3. IMU Preintegration

**Figure 2:**
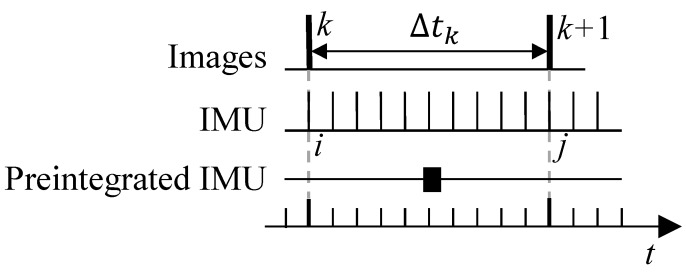
Illustration of the concept of IMU preintegration. The IMU measures the acceleration and angular velocity at discrete time. Generally, The IMU measurement frequency is much larger than the frame rate of camera. Giving two time consecutive frames $b_{k}$ and $b_{k+1}$, there exists several measurements in time interval $\left[t_{k},t_{k+1}\right]$. The IMU measurements between image frames *k* and k+1 are pre-integrated into a single compound measurement. From an optimization perspective, it can be seen as the observations or measurements with which we can construct the error terms and adjust the corresponding state values to minimize the errors.

The IMU preintegration method was firstly introduced in [35], which parameterize the rotation error using Euler angles. This method was further improved in [8] with the continuous-time quaternion-based derivation and including the handling of IMU biases. The intuitive concept of IMU preintegration is illustrated in Figure 2. The proposed an IMU preintegration method that is motivated by [8], but is different from the previous work as we introduce the IMU intrinsic parameters in the measurement model. Therefore, we need to re-derive all of the equations from scratch.

Theoretically, giving two time instants at images frames *k* and k+1, we can compute the states at $t_{k+1}$ as a function of IMU measurements between two frames:

(8) $$\begin{array}{cc} p_{b_{k+1}}^{w} & =p_{b_{k}}^{w}+v_{b_{k}}^{w}\Delta t_{k}+\frac{1}{2}g^{w}\Delta t_{k}^{2}+{\;\int \int \;}_{t\in [k,k+1]}R_{b_{t}}^{w}T_{a}\left(a_{m_{t}}-b_{a_{t}}-n_{a_{t}}\right)\;dt^{2} \end{array}$$

(9) $$\begin{array}{cc} v_{b_{k+1}}^{w} & =v_{b_{k}}^{w}+g^{w}\Delta t_{k}+\int_{t\in [k,k+1]}R_{b_{t}}^{w}T_{a}\left(a_{m_{t}}-b_{a_{t}}-n_{a_{t}}\right)\;dt \end{array}$$

(10) $$\begin{array}{cc} q_{b_{k+1}}^{w} & =q_{b_{k}}^{w}⊗\int_{t\in [k,k+1]}\frac{1}{2}q_{b_{t}}^{w}⊗T_{\omega }\left(\omega_{m_{t}}-A_{\omega }T_{a}\left(a_{m_{t}}-b_{a_{t}}-n_{a_{t}}\right)-b_{\omega_{t}}-n_{\omega_{t}}\right)\;dt, \end{array}$$

where $\Delta t_{k}$ is the duration of time interval $\left[t_{k},t_{k+1}\right]$.

While Equations (8)–(10) provide an estimation of motion between time $t_{k}$ and $t_{k+1}$, it can be seen that the IMU state propagation requires the rotation, position and velocity of frame *k* as the starting states. In the optimization-based algorithm, we adjust the full system states at every iteration step, which means these starting states may change. In such cases, we need to re-integrate the IMU measurements, which is computation consuming. To avoid this, we adopt the preintegration method.

Premultiply $q_{w}^{b_{k}}$ or $R_{w}^{b_{k}}$ to both sides of Equations (8)–(10) to change the reference frame for the IMU propagation to the local frame $b_{k}$:

(11) $$\begin{array}{cc} R_{w}^{b_{k}}p_{b_{k+1}}^{w} & =R_{b_{k}}^{w}\left(p_{b_{k}}^{w}+v_{b_{k}}^{w}\Delta t+\frac{1}{2}g^{w}\Delta t^{2}\right)+\alpha_{b_{k+1}}^{b_{k}} \end{array}$$

(12) $$\begin{array}{cc} R_{w}^{b_{k}}v_{b_{k+1}}^{w} & =R_{w}^{b_{k}}v_{b_{k}}^{w}+R_{w}^{b_{k}}g^{w}\Delta t_{k}+\beta_{b_{k+1}}^{b_{k}} \end{array}$$

(13) $$\begin{array}{cc} q_{w}^{b_{k}}⊗q_{b_{k+1}}^{w} & =\gamma_{b_{k+1}}^{b_{k}}, \end{array}$$

where

(14) $$\begin{array}{cc} \alpha_{b_{k+1}}^{b_{k}} & ={\;\int \int \;}_{t\in [k,k+1]}R_{b_{t}}^{b_{k}}T_{a}\left(a_{m_{t}}-b_{a_{t}}-n_{a_{t}}\right)\;dt^{2} \end{array}$$

(15) $$\begin{array}{cc} \beta_{b_{k+1}}^{b_{k}} & =\int_{t\in [k,k+1]}R_{b_{t}}^{b_{k}}T_{a}\left(a_{m_{t}}-b_{a_{t}}-n_{a_{t}}\right)\;dt \end{array}$$

(16) $$\begin{array}{cc} \gamma_{b_{k+1}}^{b_{k}} & =\int_{t\in [k,k+1]}\frac{1}{2}\gamma_{b_{t}}^{b_{k}}⊗T_{\omega }(\omega_{m_{t}}-A_{\omega }T_{a}\left(a_{m_{t}}-b_{a_{t}}-n_{a_{t}}\right)-b_{\omega_{t}}-n_{\omega_{t}})\;dt. \end{array}$$

The preintegration parts $\alpha_{b_{k+1}}^{b_{k}}$, $\beta_{b_{k+1}}^{b_{k}}$, $\gamma_{b_{k+1}}^{b_{k}}$ can be obtained solely with IMU measurements and its intrinsic parameters. These preintegrated measurements construct the constraints between the states at time $t_{k}$ and the states at time $t_{k+1}$. Therefore, we can construct the error terms with these preintegrated IMU measurements, and adjust the corresponding state values to minimize the errors base on optimization method.

It can be seen that the preintegration measurements in Equations (14)–(16) are also the function of the IMU intrinsic parameters, $T_{a}$, $T_{\omega }$, $A_{\omega }$ as well as the bias $b_{a}$, $b_{\omega }$. As a result, the imperfection of these parameters would introduce errors into the measurements. This also means that we can concurrently optimize the IMU intrinsic parameters to minimize the errors constrained by the preintegration measurements. To achieve this goal, two problems need to be dealt with:

1. Compute the Jacobian matrix of $\alpha_{b_{k+1}}^{b_{k}}$, $\beta_{b_{k+1}}^{b_{k}}$, $\gamma_{b_{k+1}}^{b_{k}}$ with respect to the IMU intrinsic parameters. The Jacobian matrix will be used in two ways: (1) for the optimization method, the Jacobian matrix would be used to evaluate the increment of the input arguments; (2) at the end of each iteration step, we use Jacobian as well as the new adjusted states will be used to compute the first-order approximation of preintegration measurement so that we can avoid re-integrating the IMU measurements.
2. Estimate the uncertainty of the preintegration measurements. All of the IMU measurements contain random noises. These noises, as well as the the inaccuracy of the IMU intrinsic parameters, introduce the uncertainty to the preintegration results. In order to get more precise optimization results, we need to estimate the uncertainty of the preintegration measurements in the form of a covariance matrix or information matrix and use these uncertainty information to weight the error terms during optimization.

These two problems will be tackled in the next section.

### 3.4. Jacobian and Noise Propagation

In order to compute the Jacobian matrix and estimate the uncertainty of preintegration results, we divide the true state value of preintegration result x into two components, the nominal state $\hat{x}$ and the error state δx. The nominal state is estimated by integrating the IMU measurements directly, without taking into account the noise terms and imperfect intrinsic parameters or unmodeled error. As a results, it will accumulate errors. These errors are collected into the error state. In this way, the true state can be expressed as a composition of the nominal state and error state, that is,

(17) $$\begin{array}{c} x=\hat{x}⊕\delta x, \end{array}$$

where ⊕ is the composition operator. Since the quaternion q is over-parameterized, we define its error term with the minimal representation δθ. As a result, for quaternion, ⊕ is defined as

(18) $$q=\hat{q}⊕\delta \theta \approx \hat{q}⊗\left[\begin{array}{c} 1\\ \frac{1}{2}\delta \theta \end{array}\right]\Rightarrow \delta \theta =2{\left[{\hat{q}}^{-1}⊗q\right]}_{xyz},$$

where ${\left[\cdot \right]}_{xyz}$ extracts the vector part of a quaternion q. For other states, $\hat{x}⊕\delta \hat{x}≜\hat{x}+\delta x$.

The concept of nominal-state and error-state is similar to the error-state Kalman filter (ESKF) [40,41]. The main difference is that we need to tackle the imperfection of the IMU intrinsic parameters. This is a nontrivial extension as it introduces 24 more parameters into the state space, which makes the equations more complex compared to the general ESKF and preintegration methods showed in [8,37,38].

The nominal-state is computed as follows:

(19) $$\begin{array}{cc} {\hat{\alpha }}_{b_{k+1}}^{b_{k}} & ={\;\int \int \;}_{t\in [k,k+1]}{\hat{R}}_{b_{t}}^{b_{k}}{\hat{T}}_{a}\left(a_{m_{t}}-{\hat{b}}_{a_{t}}\right)\;dt^{2} \end{array}$$

(20) $$\begin{array}{cc} {\hat{\beta }}_{b_{k+1}}^{b_{k}} & =\int_{t\in [k,k+1]}{\hat{R}}_{b_{t}}^{b_{k}}{\hat{T}}_{a}\left(a_{m_{t}}-{\hat{b}}_{a_{t}}\right)\;dt \end{array}$$

(21) $$\begin{array}{cc} {\hat{\gamma }}_{b_{k+1}}^{b_{k}} & =\int_{t\in [k,k+1]}\frac{1}{2}{\hat{\gamma }}_{b_{t}}^{b_{k}}⊗{\hat{T}}_{\omega }\left(\omega_{m_{t}}-{\hat{A}}_{\omega }{\hat{T}}_{a}\left(a_{m_{t}}-{\hat{b}}_{a_{t}}\right)-{\hat{b}}_{\omega_{t}}\right)\;dt. \end{array}$$

As the real IMU measures the acceleration and angular velocity at discrete time, Equations (19)–(21) can be computed by numberical integraion methods such as Euler, mid-point or Runge—Kutta methods. The mid-point integration is used in our implementation code, which is detailed in the Appendix A.2. At the beginning of integration, $\alpha_{b_{k}}^{b_{k}}$, $\beta_{b_{k}}^{b_{k}}$ is set to 0, and $\gamma_{b_{k}}^{b_{k}}$ is set to identity quaternion.

With the computation above, all the large-signal dynamics have been integrated in the nominal-state. As a result, the error-state is always small. We can solve its kinematics and neglect all second-order infinitesimals to get the continuous-time linearized dynamics.

(22) $$\begin{array}{cc} \delta {\dot{\alpha }}_{b_{t}}^{b_{k}} & =\delta \beta_{b_{t}}^{b_{k}} \end{array}$$

(23) $$\begin{array}{cc} \delta {\dot{\beta }}_{b_{t}}^{b_{k}} & =-{\hat{R}}_{b_{t}}^{b_{k}}{\left[{\hat{T}}_{a}\hat{a}\right]}_{\times }\delta \theta_{b_{t}}^{b_{k}}-{\hat{R}}_{b_{t}}^{b_{k}}{\hat{T}}_{a}\delta {\hat{b}}_{a}+{\hat{R}}_{b_{t}}^{b_{k}}\delta T_{a}{\hat{a}}_{B}-{\hat{R}}_{b_{t}}^{b_{k}}T_{a}n_{a} \end{array}$$

(24) $$\begin{array}{cc} \delta {\dot{\theta }}_{b_{t}}^{b_{k}}= & -{\left[{\hat{T}}_{\omega }{\hat{\omega }}_{B}\right]}_{\times }\delta \theta_{b_{t}}^{b_{k}}+{\hat{T}}_{\omega }{\hat{A}}_{\omega }{\hat{T}}_{a}\delta b_{a}-{\hat{T}}_{\omega }\delta b_{\omega }-{\hat{T}}_{\omega }{\hat{A}}_{\omega }\delta T_{a}{\hat{a}}_{B}\\ & +\delta T_{\omega }{\hat{\omega }}_{B}-{\hat{T}}_{\omega }\delta A_{\omega }T_{a}{\hat{a}}_{B}+{\hat{T}}_{\omega }{\hat{A}}_{\omega }{\hat{T}}_{a}n_{a}-{\hat{T}}_{\omega }n_{\omega }, \end{array}$$

where ${\hat{R}}_{b_{t}}^{b_{k}}$ is the corresponding rotation matrix of $\gamma_{b_{t}}^{b_{k}}$, ${\hat{a}}_{B}=\left(a_{m_{t}}-{\hat{b}}_{a_{t}}\right)$, ${\hat{\omega }}_{B}=\omega_{m_{t}}-{\hat{A}}_{\omega }{\hat{T}}_{a}\left(a_{m_{t}}-{\hat{b}}_{a_{t}}\right)-{\hat{b}}_{\omega_{t}}$, ${\left[\cdot \right]}_{\times }$ is the skew symmetry matrix operator,

(25) $${\left[\omega \right]}_{\times }=\left[\begin{array}{ccc} 0 & -\omega_{x} & \omega_{y}\\ \omega_{x} & 0 & -\omega_{z}\\ -\omega_{y} & \omega_{z} & 0 \end{array}\right].$$

Details about how to get the error-state kinematic representation above is given in the Appendix A.1.

The error-state dynamics of the IMU intrinsic parameters are as follows

(26) $$\begin{array}{cc} \delta {\dot{b}}_{a} & =n_{b_{a}},\;\delta {\dot{b}}_{\omega }=n_{b_{\omega }} \end{array}$$

(27) $$\begin{array}{cc} \delta {\dot{T}}_{a} & =0,\;\delta {\dot{T}}_{\omega }=0,\;\delta {\dot{A}}_{\omega }=0. \end{array}$$

In order to compute the the Jacobian matrix and covariance recursively, we need to convert Equations (22)–(24), (26) and (27) to matrix representation. This takes a little trick.

Define

(28) $$\begin{array}{cc} X_{pre} & =[\alpha_{b_{t}}^{b_{k}},\beta_{b_{t}}^{b_{k}},\gamma_{b_{t}}^{b_{k}},b_{a},b_{\omega },t_{a},t_{\omega },a_{\omega }], \end{array}$$

where

$$\begin{array}{cc} t_{a} & ={\left[T_{a_{11}},T_{a_{21}},T_{a_{22}},T_{a_{31}},T_{a_{32}},T_{a_{33}}\right]}^{T}\\ t_{\omega } & ={\left[T_{\omega_{11}},T_{\omega_{12}},T_{\omega_{13}},T_{\omega_{21}},T_{\omega_{22}},T_{\omega_{23}},T_{\omega_{31}},T_{\omega_{32}},T_{\omega_{33}}\right]}^{T}\\ a_{\omega } & ={\left[A_{\omega_{11}},A_{\omega_{12}},A_{\omega_{13}},A_{\omega_{21}},A_{\omega_{22}},A_{\omega_{23}},A_{\omega_{31}},A_{\omega_{32}},A_{\omega_{33}}\right]}^{T}. \end{array}$$

With $T_{a_{ij}}$, $T_{\omega_{ij}}$, $A_{\omega_{ij}}$ are the corresponding elements of the matrix $T_{a}$, $T_{\omega }$, $A_{\omega }$, respectively.

Assume $r_{3\times 1}={\left[r_{1},r_{2},r_{3}\right]}^{T}$. Now we define the ${\left[\cdot \right]}_{T}$ operator as

$${\left[r_{3\times 1}\right]}_{T}={\left[\begin{array}{cccccc} r_{1} & 0 & 0 & 0 & 0 & 0\\ 0 & r_{1} & r_{2} & 0 & 0 & 0\\ 0 & 0 & 0 & r_{1} & r_{2} & r_{3} \end{array}\right]}_{3\times 6},\;\mathrm{such}\;\mathrm{that}\;T_{a}r_{3\times 1}={\left[r_{3\times 1}\right]}_{T}t_{a}$$

$$\mathrm{and}\;{\left[r_{3\times 1}\right]}_{T}={\left[\begin{array}{ccc} r^{T} & 0 & 0\\ 0 & r^{T} & 0\\ 0 & 0 & r^{T} \end{array}\right]}_{3\times 9},\;\mathrm{such}\;\mathrm{that}\;T_{\omega }r_{3\times 1}={\left[r_{3\times 1}\right]}_{T}t_{\omega },A_{\omega }r_{3\times 1}={\left[r_{3\times 1}\right]}_{T}a_{\omega }.$$

With the definitions above, Equations (22)–(24), (26) and (27) can be converted to matrix representation form

(29) $$\begin{array}{c} \delta {\dot{X}}_{pre}=F_{t}\delta X_{pre}+G_{t}n \end{array}$$

where

$$F_{t}=\left[\begin{array}{c} \begin{array}{c} \begin{array}{cccccccc} 0 & I & 0 & 0 & 0 & 0 & 0 & 0\\ 0 & 0 & -{\hat{R}}_{b_{t}}^{b_{k}}{\left[{\hat{T}}_{a}\hat{a}\right]}_{\times } & -{\hat{R}}_{b_{t}}^{b_{k}}{\hat{T}}_{a} & 0 & {\hat{R}}_{b_{t}}^{b_{k}}{\left[{\hat{a}}_{t}\right]}_{T} & 0 & 0\\ 0 & 0 & {\left[{\hat{T}}_{\omega }{\hat{\omega }}_{t}\right]}_{\times } & {\hat{T}}_{\omega }{\hat{A}}_{\omega }{\hat{T}}_{a} & {\hat{T}}_{\omega } & -{\hat{T}}_{\omega }{\hat{A}}_{\omega }{\left[{\hat{a}}_{t}\right]}_{T} & {\left[\hat{\omega }\right]}_{T} & {\hat{T}}_{\omega }{\left[{\hat{T}}_{a}{\hat{a}}_{t}\right]}_{T} \end{array}\\ 0_{30\times 39} \end{array} \end{array}\right]$$

$$G_{t}=\left[\begin{array}{c} \begin{array}{c} \begin{array}{cccc} 0 & 0 & 0 & 0\\ {\hat{T}}_{\omega }{\hat{A}}_{\omega }{\hat{T}}_{a} & -{\hat{R}}_{b_{t}}^{b_{k}}{\hat{T}}_{a} & 0 & 0\\ 0 & -{\hat{T}}_{\omega } & 0 & 0\\ 0 & 0 & I & 0\\ 0 & 0 & 0 & I \end{array}\\ 0_{24\times 12} \end{array} \end{array}\right],$$

and $n=[n_{a},n_{\omega },n_{b_{a}},n_{b_{\omega }}]$.

With the Equation (29), it is convenient to compute the the covariance matrix $P_{b_{k+1}}^{b_{k}}$ recursively by the first order discrete-time covariance update

(30) $$P_{b_{t+\delta t}}^{b_{k}}=\left(I+F_{t}\delta t\right)P_{b_{t}}^{b_{k}}{\left(I+F_{t}\delta t\right)}^{T}+\left(G_{t}\delta t\right)Q{\left(G_{t}\delta t\right)}^{T},$$

where δt is the time between two IMU measurements, and Q is the diagonal covariance matrix of noise $\left(\sigma_{a}^{2},\sigma_{g}^{2},\sigma_{b_{a}}^{2},\sigma_{b_{g}}^{2}\right)$. The initial covariance value corresponding to α, β, γ, $b_{a}$, $b_{\omega }$ is set to 0. For $T_{a}$, $T_{\omega }$, $A_{\omega }$, the initial covariance can be set to 0 or a constant uncertainty, which will be discussed in Section 3.6.

Now we deal with the Jacobian matrix. Neglecting the noise term, Equation (29) can be converted to a discrete-time representation as following:

(31) $$\begin{array}{c} \delta X_{pre}\left(t+\delta t\right)=\left(1+F_{t}\delta t\right)\delta X_{pre}\left(t\right). \end{array}$$

As a result, the Jacobian of $\delta X_{pre}\left(t+\delta t\right)$ with respect to $\delta X_{pre}\left(t\right)$ can be obtained conveniently:

(32) $$J_{t}^{t+\delta t}=\frac{\partial \delta {\partial X}_{pre}\left(t+\delta t\right)}{\delta X_{pre}\left(t\right)}=1+F_{t}\delta t.$$

We can compute $J_{k}^{k+1}$ recursively:

(33) $$\begin{array}{cc} J_{k}^{k+1} & =\frac{\partial \delta X_{pre}\left(j\right)}{\partial \delta X_{pre}\left(j-1\right)}\frac{\partial \delta X_{pre}\left(j-1\right)}{\partial \delta X_{pre}\left(j-2\right)}\cdots \frac{\partial \delta X_{pre}\left(i+1\right)}{\partial \delta X_{pre}\left(i\right)}\\ & =\left(I+F_{t,j-1}\delta t_{j-1}\right)\left(I+F_{t,j-2}\delta t_{j-2}\right)\cdots \left(I+F_{i}\delta t_{i}\right), \end{array}$$

where *i* and *j* represent the *i*-th and *j*-th IMU measurement obtained at the time instant of frame *k* and k+1, respectively, as illustrated in Figure 2.

The first order approximation of $\alpha_{b_{k+1}}$, $\beta_{b_{k+1}}$, $\gamma_{b_{k+1}}$ with respect to the IMU intrinsic parameters can be computed as:

(34) $$\begin{array}{cc} \alpha_{b_{k+1}}^{b_{k}} & ={\hat{\alpha }}_{b_{k+1}}^{b_{k}}+J_{b_{a}}^{\alpha }\delta b_{a_{k}}+J_{b_{\omega }}^{\alpha }\delta b_{\omega_{k}}+J_{t_{a}}^{\alpha }\delta t_{a_{k}}+J_{t_{\omega }}^{\alpha }\delta t_{\omega_{k}}+J_{a_{\omega }}^{\alpha }\delta a_{\omega_{k}} \end{array}$$

(35) $$\begin{array}{cc} \beta_{b_{k+1}}^{b_{k}} & ={\hat{\beta }}_{b_{k+1}}^{b_{k}}+J_{b_{a}}^{\beta }\delta b_{a_{k}}+J_{b_{\omega }}^{\beta }\delta b_{\omega_{k}}+J_{t_{a}}^{\beta }\delta t_{a_{k}}+J_{t_{\omega }}^{\beta }\delta t_{\omega_{k}}+J_{a_{\omega }}^{\beta }\delta a_{\omega_{k}} \end{array}$$

(36) $$\begin{array}{cc} \gamma_{b_{k+1}}^{b_{k}} & ={\hat{\gamma }}_{b_{k+1}}^{b_{k}}⊗(J_{b_{a}}^{\gamma }\delta b_{a_{k}}+J_{b_{\omega }}^{\gamma }\delta b_{\omega_{k}}+J_{t_{a}}^{\gamma }\delta t_{a_{k}}+J_{t_{\omega }}^{\gamma }\delta t_{\omega_{k}}+J_{a_{\omega }}^{\gamma }\delta a_{\omega_{k}}), \end{array}$$

where $J_{b_{a}}^{\alpha }$ is the sub-block matrix of $J_{b_{k+1}}$ whose location is corresponding to $\frac{\partial \delta \alpha_{b_{k+1}}^{b_{k}}}{b_{a_{k}}}$. The same meaning is also used for other J in the above.

At each step of optimization, we can use Equations (34)–(36) to update the value of α, β, γ with the new estimated IMU intrinsic parameters and recompute the corresponding residuals.

### 3.5. IMU Measurement Factor

Considering two consecutive frames *k* and k+1 in the window, the residual of preintegrated IMU measurement can be constructed from Equations (11)–(13):

(37) $$r_{B}\left({\hat{z}}_{b_{k+1}}^{b_{k}},X\right)=\left[\begin{array}{c} \delta {\hat{\alpha }}_{b_{k+1}}^{b_{k}}\\ \delta {\hat{\beta }}_{b_{k+1}}^{b_{k}}\\ \delta {\hat{\gamma }}_{b_{k+1}}^{b_{k}}\\ \delta b_{a}\\ \delta b_{\omega } \end{array}\right]=\left[\begin{array}{c} R_{w}^{b_{k}}\left(p_{b_{k+1}}^{w}-p_{b_{k}}^{w}+\frac{1}{2}g^{w}\Delta t^{2}-v_{b_{k}}^{w}\Delta t\right)-{\hat{\alpha }}_{b_{k+1}}^{b_{k}}\\ R_{w}^{b_{k}}\left(v_{b_{k+1}}^{w}+g^{w}\Delta t-v_{b_{k}}^{w}\right)-{\hat{\beta }}_{b_{k+1}}^{b_{k}}\\ 2{\left[{\left({\hat{\gamma }}_{b_{k+1}}^{b_{k}}\right)}^{-1}⊗{\left(q_{b_{k}}^{w}\right)}^{-1}⊗q_{b_{k+1}}^{w}\right]}_{xyz}\\ b_{a,k+1}-b_{a,k}\\ b_{\omega ,k+1}-b_{\omega ,k} \end{array}\right].$$

Please note that the accelerometer and gyroscope bias are also included in the residual terms for online correction. Equation (37) seems the same as the IMU measurements residual in the paper [8]. However, as our preintegrated IMU measurements α, β, γ handle the imperfection of the IMU intrinsic parameters, which made it different from the one of [8]. Besides, the Jacobian of $r_{B}\left({\hat{z}}_{b_{k+1}}^{b_{k}},X\right)$ with respect to the input arguments is completely different, too.

### 3.6. IMU Intrinsic Factor

There are several possible but different strategies for defining the IMU intrinsic parameters residual:

1. The parameters $T_{a}$, $T_{\omega }$, $A_{\omega }$ are modeled as random-walk processes, but are constant between two frames in the sliding window, and are handled with the same strategy as the IMU bias.
2. The parameters $T_{a}$, $T_{\omega }$, $A_{\omega }$ are assumed to be uncertain but constant between two frames in the sliding window. With this strategy, we can set the covariance value of $P_{b_{k}}^{b_{k}}$ in Equation (30) corresponding to $T_{a}$, $T_{\omega }$, $A_{\omega }$ with a constant value and optimize them at every frame.
3. The parameters $T_{a}$, $T_{\omega }$, $A_{\omega }$ are assumed to be uncertain, but constant in the timespan of the sliding window.

From the perspective of optimization method, the strategies 1 and 2 are nearly same, except that the covariance value is different, with strategy 1 computing the covariance during preintegration. However, for every frame these two strategies would add additional 24 parameters to the estimator. Assuming the size of sliding window is 10, this means that we will need to optimize an additional 240 parameters at each step, leading to a computational problem for realtime implementation. Besides, compared to the accelerometer and gyroscope bias which vary with time and temperature, the scale factor and misalignment parameters seem more stable, which means that we do not need to optimize them at every frame. Moreover, the scale factors and axis misalignments may couple with the measurement bias under degenerate motions such as rectilinear trajectory, motion with zero acceleration or rotation. In such a case, it is impossible to distinguish all these parameters in one or two frames. With these reasons, in our algorithm, we would choose the strategy 3.

The residual of $T_{a}$, $T_{\omega }$, $A_{\omega }$ in our implementation is defined as follows:

(38) $$r_{I}\left(X\right)=\left[\begin{array}{c} t_{a}-{\hat{t}}_{a,k}\\ t_{\omega }-{\hat{t}}_{\omega ,k}\\ a_{\omega }-{\hat{a}}_{\omega ,k} \end{array}\right],$$

where ${\hat{t}}_{a,k}$, ${\hat{t}}_{\omega ,k}$ and ${\hat{a}}_{\omega ,k}$ are the current estimation of IMU intrinsic parameters. Please note that the residual is weighted by a constant covariance matrix $P_{I}$ and add to the cost function, Equation (2).

### 3.7. Discussion and Implementation Details

The considered IMU intrinsic parameters in our method include axis misalignments, scale factors and g-sensitivity. In practice, however, it should be noted that online g-sensitivity calibration is a difficult task. This my be caused by various reasons. First, the errors caused by g-sensitivity are generally smaller than other errors, especially in cases that the IMU suffers from motion with low acceleration or constant speed, or remains static. Second, the g-sensitivity coefficients of a real IMU may vary with the frequency of vibration. As a result, the errors caused by g-sensitivity are more prone to couple with the gyroscope bias or manifest as random noise. In some worse cases, online g-sensitivity calibration may lead to less accuracy of the motion estimation of VINS. Moreover, g-sensitivity calibration would introduce an extra nine variables to the state space, which would consume more computational resource. Therefore, in practice application, whether or not calibrating the g-sensitivity online depends on the IMU used and the motion that the sensor system are subjected. Fortunately, it is very easy to calibrate without g-sensitivity by setting the g-sensitivity coefficients to zeros and keep them constant. The implementation details would be discussed in the following.

**Figure 3:**
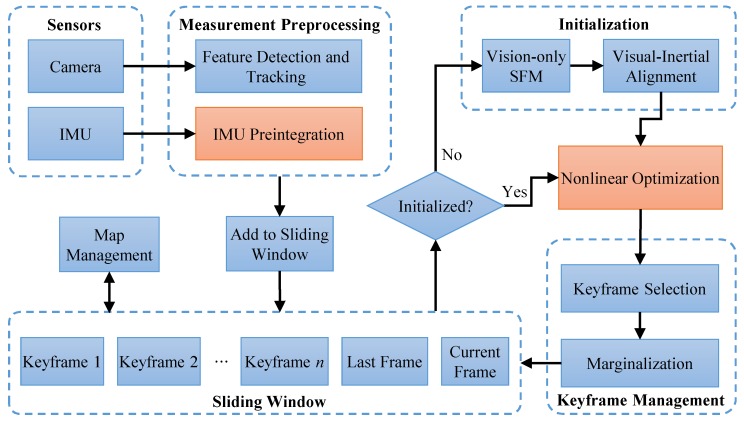
Block diagram illustrating of a monocular visual-inertial system (VINS-Mono) [8], based on which, we implement the proposed online IMU self-calibration method. We add the IMU intrinsic parameters into the state vector and replace the IMU Preintegration block with our method, which are detailed in Section 3.3 and Section 3.4. The proposed IMU measurement factor (Section 3.5) and IMU intrinsic factor (Section 3.6) are used in the nonlinear optimization block.

We implemented the proposed online IMU self-calibration method with the extension of VINS-Mono [8], as depicted in Figure 3. The system started with measurement preprocessing. For each new image, features were detected by the Shi–Tomasi corner detector [42] and tracked by a KLT tracker [43]. Meanwhile, IMU measurements were locally pre-integrated. The initialization procedure provided all necessary values for bootstrapping the subsequent nonlinear optimization. After the system has been successfully initialized, the tightly-coupled nonlinear optimization would be implemented when every frame is added to the sliding window. After that, keyframe management module marginalized one of the frames from the sliding window in order to bound the computation complexity. We add the IMU intrinsic parameters into the state vector and replace the IMU preintegration block with our method, which are detailed in Section 3.3 and Section 3.4. The proposed IMU measurement factor (Section 3.5) and IMU intrinsic factor (Section 3.6) were used in the nonlinear optimization block. The optimization computation is based on the Ceres Solver [44]. In the implementation, we separate the $T_{a}$, $T_{\omega }$, $A_{\omega }$ into individual parameter blocks so that we can conveniently set any blocks to constant when optimization, depending the real application. In such way, we can implement our method with and without g-sensitivity calibration, as discussed above.

## 4. Experimental Results

We performed dataset experiments to evaluate the proposed online IMU self-calibration method. At present there are several publicly available datasets for VINS evaluation, such as UMich NCLT [45], EuRoc [46], PennCOSYVIO [47], Zurich Urban MAV [48], TUM VI [49], etc., in which, EuRoc is a very popular dataset and used by many papers [8,20,38,39,50,51,52]. For a completely comparison of these datasets, please refer to [49]. In this paper we adopt the TUM VI dataset [49] for two main reasons: (1) the IMU used in EuRoc dataset is an ADIS16448, a high-end industrial grade IMU with precisely factory calibrated. In contrast, the TUM VI uses a low-cost consumer grade IMU, Bosch BMI160, which makes it more suitable for evaluating our algorithm; (2) The dataset provides both the raw IMU measurements and the precalibrated IMU measurements that compensated for proper IMU scaling and axis alignment. The value of scale factor and axis alignment parameters used for compensation are also provided with the dataset. As a result, we can easily run our algorithm with the raw IMU measurements and compare the estimated intrinsic parameters with the provided reference value.

The TUM VI dataset provides several categories of sequences for the evaluation of VINS method, including three corridor sequences, six magistrale sequences, eight outdoor sequences, six room sequences, and three slider sequence. The outdoor sequences are neglected in our experiments as we focus on the IMU self-calibration but not long time drift evaluation in challenging environments. To perform the comparison experiments, we firstly packed the the quarter resolution images (512×512 pixels) of the left camera, precalibred IMU measurements as well as the raw IMU measurements with different topic names into one single ROS bag file for each sequence so that we can easily evaluate the algorithm with different type of IMU measurements by just modifying the topic name.

Please note that we deduct the large IMU bias, which is coarsely reproducible between sensor restarts, from the raw measurements. This is reasonably explained in [49]. We also compensate the timestamp of IMU data so that the timestamp is consistent for camera, IMU and ground truth pose.

### 4.1. IMU Intrinsics Estimation Results

As discussed in Section 3.7, it is difficult to calibrate the g-sensitivity online. In the experiments, we ran the algorithm two times on each sequence of dataset, with and without $A_{\omega }$ estimation, respectively. Then we compared the IMU intrinsics estimation results with the the reference values. In all experiments, the initial value of $T_{a}$ and $T_{\omega }$ were set to the identity matrix, with the covariance as $2.0\times 10^{-4}$, and the initial value $A_{\omega }$ was set as a zeros matrix with covariance as $5.0\times 10^{-6}$.

#### 4.1.1. IMU Intrinsics Estimation without G-Sensivity

**Figure 4:**
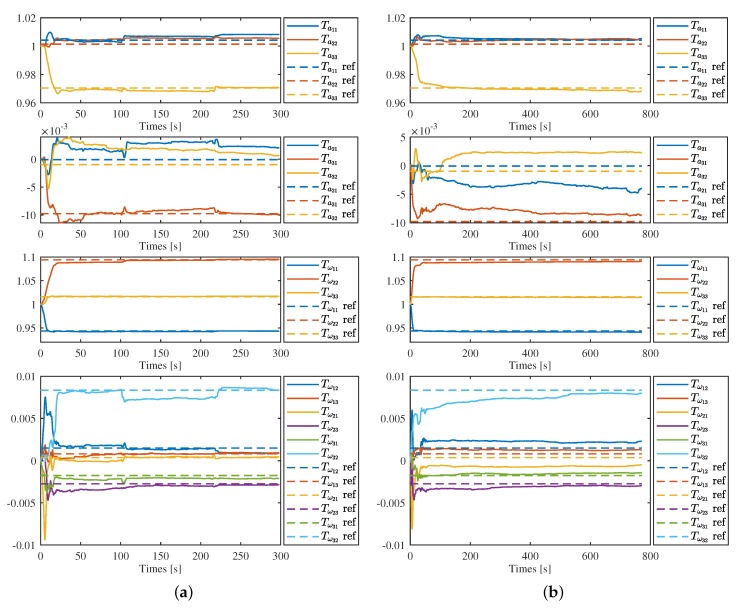
IMU intrinsics estimation in corridor 1 (**a**) and magistrale 1 (**b**). Solid lines are the estimation results and the dotted lines with the same color are the corresponding reference value.

**Figure 5:**
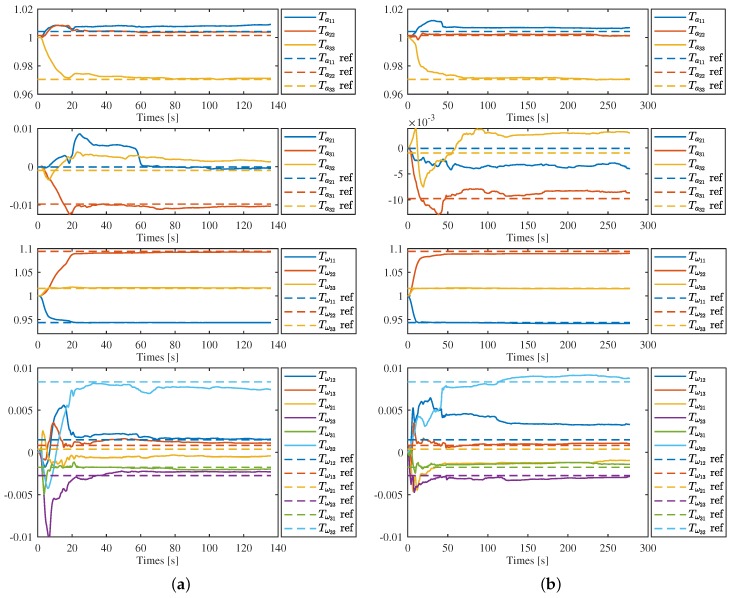
IMU intrinsics estimation in room 1 (**a**) and slider 1 (**b**). Solid lines are the estimation results and the dotted lines with the same color are the corresponding reference value.

Figure 4 and Figure 5 show the estimation results on the first sequence of each categories, corridor 1, magistrale 1, room 1, slides 1, respectively, in detail. We can see that all parameters converge to a certain range close to the ref value, but slightly vary with time after that. For each sequence, the convergence speed is different. Generally, the parameters of the gyroscope converge in about 15–20 s and the estimation results is more stable. In contrast, it takes 50–100 s for the parameters of accelerometer to converge. In Figure 4b and Figure 5b, the estimation results of $T_{a_{21}}$ and $T_{a_{32}}$ show larger differences compared with the results of Figure 4a and Figure 5a.

It is not surprising for the results mentioned above, as many factors would affect the accuracy of the estimation results. Firstly, the VINS problem has the characteristics of high nonlinearity and local weak observability. To achieve full observability of the IMU intrinsic parameters as well as the measurement bias, motion with sufficient excitation was required. It is certain that this condition would not be always satisfied at any time in real applications. Therefore, the result shows fluctuation under insufficient excitation motion. Besides, the convergence speeds were affected by different motions, which shows a different convergence speed for each sequence. Secondly, as the accelerometer measures the acceleration and the gyroscope can measure the angular velocity directly, this also means that different conditions are required for the accelerometer intrinsics and gyroscope intrinsics to achieve observability. For the accelerometer, sufficient acceleration on each axis is required, and for the gyroscope, only sufficient rotation of the system is required. For a moving sensing system, keeping sufficient acceleration all the time is infeasible in real applications. In contrast, keeping sufficient rotation is a more easily satisfied condition. As a result, the convergence speed of gyroscope shows faster and more stable than that of accelerometer. Thirdly, the measurements of vision sensor and IMU are affected by random noise, which is coupled with the erros caused by inaccuracy intrinsics and bias, and further affects the accuracy of the estimation results. Generally, the measurement noise of gyroscopes is much smaller than that of accelerometers, which means the estimated results of gyroscopes are more accurate than accelerometers.

From Figure 4 and Figure 5, it seems that the estimation results of intrinsic parameters located at the diagonal of matrix $T_{a}$ and $T_{\omega }$ are more stable and accurate than other parameters located at the lower or upper triangle. This is because parameters of the lower or upper triangle themselves are much smaller than those of diagonal, and any small challenges would show large fluctuations in the figures. To further evaluate the estimate results, we take the estimation values at the end of each sequence as the final estimation results and calculate their mean value, standard deviation as well as the differences compared with the corresponding reference values. The statistics are shown in the Table 1 and Table 2. We can see that: (1) the standard deviation of diagonal parameters and that of off-diagonal parameters do not show obvious differences; (2) The standard deviation of gyroscope intrinsics is much smaller than that of accelerometer intrinsics. In Table 1, the standard deviation of results was about 0.002. In contrast, five of nine parameters’ standard deviations in Table 2 were less than 0.001. This means that the results of the gyroscope were more accurate, which concludes the analysis above. Generally, the axis misalignments of low-cost MEMS-based IMU was 0.5–1%, and the standard deviations of our estimation results was in the order of magnitude $10^{-3}$. Therefore, we can conclude that the proposed method can works well with the low-cost MEMS-based IMU.

#### 4.1.2. IMU Intrinsics Estimation with G-Ensitivity

**Figure 6:**
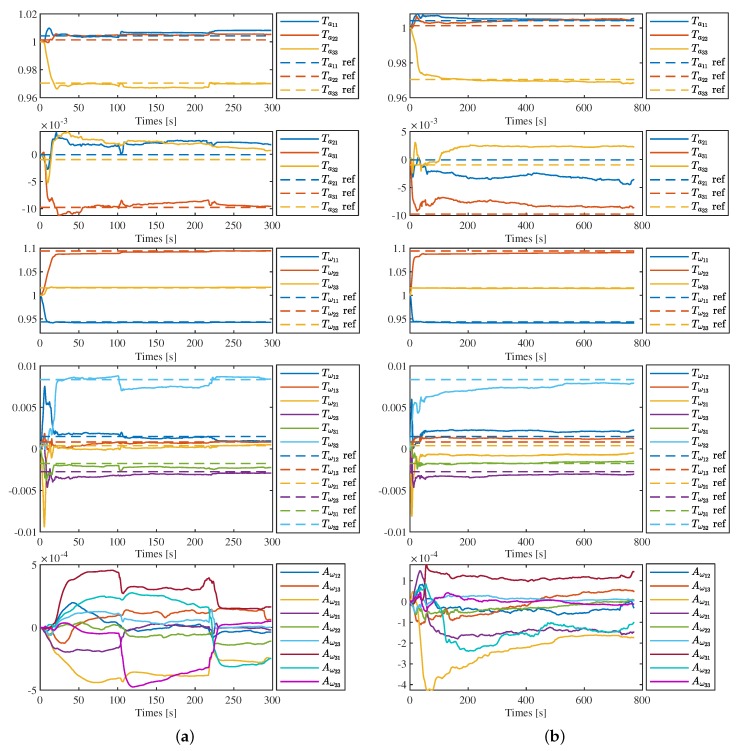
IMU intrinsics estimation with g-sensitivity in corridor 1 (**a**) and magistrale1 (**b**). Solid lines are the estimation results and the dot lines with the same color are the corresponding reference value.

As the g-sensitivity coefficients are unknown, here we only show the results of sequence of corridor 1 and magistrale 1 in detail, as in Figure 6a,b. Comparing these two figures with the estimation results without g-sensitivity showed in Figure 4a,b, we can find that the reuslts of $T_{a}$ and $T_{\omega }$ are nearly same, regardless of with or without g-sensivity estimation. The results of g-sensivity $A_{\omega }$ do not show that they are very stable. However, this does not mean that we obtained the wrong estimations, or that the calibration of g-sensitivity is totally failed. We would further discuss this in the next section with the quantitative results of VINS to indirectly evaluation to g-sensitivity estimation.

### 4.2. Quantitative Evaluation

**Figure 7:**
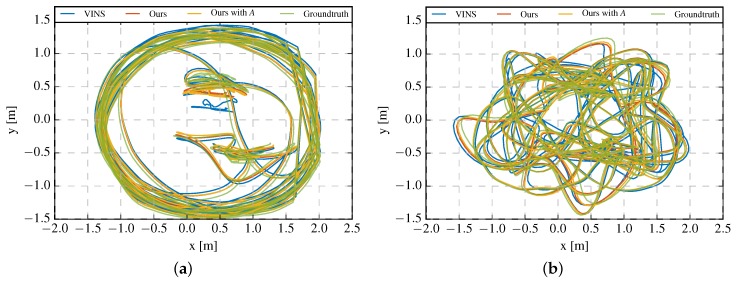
Trajectory of (**a**) rooms 3 and (**b**) room 5.

The goal of the IMU calibration is to estimate the IMU intrinsics and compensate the measurements of IMU, and to improve the performance of VINS. Therefore, the accuracy of the IMU intrinsics calibration would directly affects the results of motion estimation. In this section, we quantify the accuracy of the estimation result of system pose with absolute trajectory error (ATE) and relative pose error (RPE) [53]. The RPE is only available for the sequences of rooms 1–6, as for other sequences only accurate pose ground truth at the start and end are available. For comparison, we also ran the VINS-Mono with the same camera image sequences and configuration (IMU noise, camera intrinsics, etc.), but with the precalibrated IMU measurements provided by the dataset. We did not run VINS-Mono directly with the raw IMU measurements as it fails after a few seconds on all sequences. For each sequence, we run the proposed algorithm and the VINS-Mono five times. In each run of the proposed algorithm, the IMU intrinsic parameters estimated online are saved after each nonlinear optimization step and used as the initial values of the next run. The median values obtained by the five times running were used as the final result. The trajectory estimation results of sequence rooms 3 and 5 are showed in Figure 7. To simplify the notation, in the following figures and tables, we use VINS to denote the results of VINS-Mono, our results and our results with $A_{\omega }$ to denote the results of our proposed method without and with g-sensitivity estimation, respectively.

The root-mean-square-error (RMSE) ATE of all sequences is shown in Table 3. Compared the results of our method without g-sensitivity estimation (column 3) against those of VINS-Mono (column 2), 13/20 of the our results method show improvement performance, with the rest of results offering near performance to those of VINS-Mono except the sequence magistrales 3 and 6. What interested us is that, although the estimation results of $T_{a_{21}}$ and $T_{a_{32}}$ in sequence magistrale 1 and slider 1 show larger differences compared to the reference values, the ATE of these two sequences show obvious improvement. This means there exist some errors in the precalibrated reference values or the intrinsic parameter values vary with time, as we mentioned in Section 1. Therefore, our VINS implementaion with online IMU self-calibration shows superior performance compared against the result of VINS-Mono with offline precalibrated IMU measurements.

Now we further focus on the ATE results of our method with g-sensitivity estimation (column 4). 9/20 of the results show best performance between the three algorithms, most of which show more than 50% improvements. However, some of the results show worse performance compared to the estimation results without g-sensitivity calibration. This also concludes our discussion in Section 3.7 that online g-sensitivity calibration is a difficult task and we would not always get a better performance with g-sensitivity estimation.

**Figure 8:**
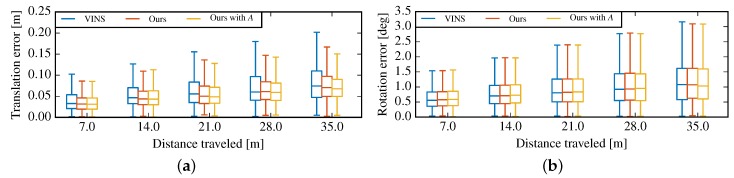
Overall relative pose error in rooms 1–6. (**a**) Relative translation error; (**b**) Relative orientation error

Figure 8 shows the overall relative pose error (RTE) in room1–room6, caculated by the toolbox [54]. In Figure 8a, we can find obvious improvements compared to VINS-Mono. In contrast, nearly same relative orientation errors are showed in Figure 8b.

### 4.3. Runtime Evaluation

**Figure 9:**
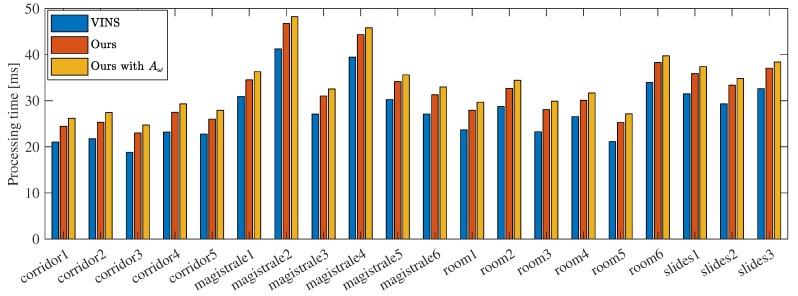
Average processing time per frame.

The proposed online IMU self-calibration method adds additional states to the system state vector, and estimates the optimal value of these states based on optimization method. As a result, online calibration would consume more computation resource. Figure 9 shows the average processing time per frame on each sequence. All experiments were performed using a computer equipped with an Intel Core(TM) i7-7700 CPU at 3.60 GHz and 8 GB RAM.

It is obvious that the proposed method consumes an additional 3–5 ms to estimate the IMU intrinsics. We further compute the average processing time for all sequences and the results are 27.7 ms, 31.8 ms, 33.5 ms, respectively. The processing time of our method without and with g-sensitivity estimation increase 14.9% and 20.9%, respectively, compared with VINS-Mono. Nevertheless, we can conclude that our method can run in real time.

Calibration process with g-sensitivity would introduce extra nine variables to the state space and consumes about additional 2 ms times the cost. Therefore, in practice applications, it is necessary to decide whether or not calibrating the g-sensitivity online, according to the IMU used and the trade-off between estimation accuracy and real time performance. Beside, the motion of the sensing system that are subjected should be considered, too. For example, if the general motion of the system is in low acceleration most of the time, g-sensitivity calibration may not significantly improve the performance of VINS.

## 5. Conclusions

In this paper, we propose an online IMU self-calibration method for visual-inertial system with low-cost inertial sensors. The main advantage of our method is that it performs online IMU self-calibration based on optimization method in unknown environment, using only camera and inertial measurements, and without any knowledge about the structure of scene or any external equipment. Specifically, the estimated parameters include the axis misalignments and scale factors of the IMU, as well as the g-sensitivity of the gyroscope. We perform extensive evaluation on public TUM VI dataset, with and without g-sensitivity estimation, respectively. Experimental results verify that the proposed method is able to accurately calibrate all the considered parameters in real time, and show superior performance compared with the results of VINS with offline precalibrated IMU measurements. It should be noted that the calibration process with g-sensitivity would introduce extra nine variables to the state space and consume more computation resource. Therefore, in practical application, it is necessary to decide whether or not calibrating the g-sensitivity online, according to the IMU used and the trade-off between estimation accuracy and real time performance.

The proposed method is dedicated to low-cost IMUs. As a result, it is very suitable for the consumer grade mobile robots, drones and cellphones which are broadly equipped with low-cost MEMS-based IMUs. Moreover, our method would promote the rapid deployment of VINS in various applications base on these platforms, such as robot navigation, autonomous driving, AR and VR.

In this paper we adopt quaternion as global parameterization for rotations. In the future we will extend our method to work with the special orthogonal group SO(3), which would make it more easily be employed in existing SO(3)-based visual/visual-inertial frameworks.

## Figures and Tables

**Table 1:** Statistics of the estimation results of accelerometer intrinsics.

	$T_{a_{11}}$	$T_{a_{21}}$	$T_{a_{22}}$	$T_{a_{31}}$	$T_{a_{32}}$	$T_{a_{33}}$
Reference	1.0042	−0.0001	1.0014	−0.0098	−0.0010	0.9705
Mean	1.0060	−0.0019	1.0040	−0.0090	0.0006	0.9700
Standard deviations	0.0017	0.0024	0.0020	0.0010	0.0028	0.0021
|Mean-reference|	0.0018	0.0018	0.0026	0.0008	0.0016	0.0004

**Table 2:** Statistics of the estimation results of gyroscope intrinsics.

	$T_{\omega_{11}}$	$T_{\omega_{12}}$	$T_{\omega_{13}}$	$T_{\omega_{21}}$	$T_{\omega_{22}}$	$t_{\omega_{23}}$	$t_{\omega_{31}}$	$t_{\omega_{32}}$	$t_{\omega_{33}}$
Reference	0.9436	0.0015	0.0008	0.0004	1.0941	−0.0027	−0.0018	0.0083	1.0159
Mean	0.9416	0.0023	0.0009	−0.0005	1.0919	−0.0012	−0.0015	0.0064	1.0160
Standard deviations	0.0009	0.0009	0.0004	0.0006	0.0018	0.0022	0.0004	0.0030	0.0012
|Mean-reference|	0.0020	0.0008	0.0000	0.0009	0.0022	0.0015	0.0003	0.0019	0.0002

**Table 3:** Root-mean-square-error (RMSE) absolute trajectory error (ATE) comparison between the proposed method with raw inertial measurement unit (IMU) measurements and monocular visual-inertial system (VINS-Mono) with precalibrated IMU measurements in all of the sequences. Downarrow means better performance compared to VINS-Mono. The bold values indicate the best results.

Sequence	VINS	Ours	Ours with $A_{\omega }$	Length (m)
corridor1	0.618	**0.453 ↓**	0.851	305
corridor2	1.174	1.157 ↓	**0.936 ↓**	322
corridor3	1.309	0.797 ↓	**0.447 ↓**	300
corridor4	0.309	0.195 ↓	**0.136 ↓**	114
corridor5	0.674	**0.547 ↓**	0.647 ↓	270
magistrale1	2.385	2.043 ↓	**1.072 ↓**	918
magistrale2	3.205	**3.105 ↓**	3.132 ↓	561
magistrale3	**0.358**	0.521	1.035	566
magistrale4	4.443	**3.919 ↓**	4.223 ↓	688
magistrale5	**0.542**	0.603	0.738	458
magistrale6	2.078	2.825	**1.210 ↓**	771
room1	**0.089**	0.095	0.094	146
room2	**0.048**	0.051	0.049	142
room3	0.145	**0.076 ↓**	0.084 ↓	135
room4	**0.042**	0.048	0.052	68
room5	0.196	0.049 ↓	**0.043 ↓**	131
room6	0.059	0.057 ↓	**0.048 ↓**	67
slides1	0.517	0.273 ↓	**0.193 ↓**	289
slides2	**1.007**	1.047	1.209	299
slides3	1.005	0.781 ↓	**0.764 ↓**	383

## Abbreviations

The following abbreviations are used in this manuscript:

IMU	Inertial measurement unit
MEMS	Microelectro mechanical systems
AHRS	Attitude and heading reference system

## Appendix A. Quaternion-Based IMU Preintegration

### Appendix A.1. The Error-State Kinematics

This section details how to obtain the the error-state kinematic in Section 3.4. Our goal is to determine $\delta \dot{\alpha }$, $\delta \dot{\beta }$, $\delta \dot{\gamma }$. For readable and simplicity purpose, we will drop the coordinate frame subscripts in the following equations, which would not cause ambiguous.

From Equations (14)–(16), we can get the true state kinematics of preintegration:

(A1) $$\begin{array}{cc} \dot{\alpha } & =\beta \end{array}$$

(A2) $$\begin{array}{cc} \dot{\beta } & =RT_{a}\left(a_{m}-b_{a}-n_{a}\right) \end{array}$$

(A3) $$\begin{array}{cc} \dot{\gamma } & =\frac{1}{2}\gamma ⊗T_{\omega }(\omega_{m}-A_{\omega }T_{a}\left(a_{m}-b_{a}-n_{a}\right)-b_{\omega }-n_{\omega }). \end{array}$$

From Equations (19)–(21), the nominal-states kinematic is obtained as follows:

(A4) $$\begin{array}{cc} \dot{\hat{\alpha }} & =\hat{\beta } \end{array}$$

(A5) $$\begin{array}{cc} \dot{\hat{\beta }} & =\hat{R}{\hat{T}}_{a}\left(a_{m}-{\hat{b}}_{a}\right) \end{array}$$

(A6) $$\begin{array}{cc} \dot{\hat{\gamma }} & =\frac{1}{2}\hat{\gamma }⊗{\hat{T}}_{\omega }\left(\omega_{m}-{\hat{A}}_{\omega }{\hat{T}}_{a}\left(a_{m}-{\hat{b}}_{a}\right)-{\hat{b}}_{\omega }\right). \end{array}$$

A. The Translation Error

With Equations (A3) and (A6), it is easy to obtain:

(A7) $$\delta \dot{\alpha }=\beta -\hat{\beta }=\delta \beta$$

B. The Linear Velocity Error

We start with the following relations:

(A8) $$\begin{array}{cc} R & =\hat{R}\left(I+{\left[\delta \theta \right]}_{\times }\right)+O\left({∥\delta \theta ∥}^{2}\right) \end{array}$$

(A9) $$\begin{array}{cc} a & =T_{a}\left(a_{m}-b_{a}-n_{a}\right)\\ & ={\hat{T}}_{a}{\hat{a}}_{B}+{\hat{T}}_{a}\delta a_{B}+\delta {\hat{T}}_{a}{\hat{a}}_{B}+\delta {\hat{T}}_{a}\delta a_{B}, \end{array}$$

where ${\hat{a}}_{B}=a_{m}-b_{a}$, $\delta a_{B}=-\delta b_{a}-n_{a}$.

Substituting Equations (A8) and (A9) into Equations (A2) and (A5) yields:

$$\begin{array}{cc} \dot{\beta } & \approx \hat{R}\left(I+{\left[\delta \theta \right]}_{\times }\right)\left({\hat{T}}_{a}{\hat{a}}_{B}+{\hat{T}}_{a}\delta a_{B}+\delta {\hat{T}}_{a}{\hat{a}}_{B}+\delta {\hat{T}}_{a}\delta a_{B}\right)\\ & =\hat{R}{\hat{T}}_{a}{\hat{a}}_{B}+\hat{R}{\hat{T}}_{a}\delta a_{B}+\hat{R}\delta {\hat{T}}_{a}{\hat{a}}_{B}+\hat{R}\delta {\hat{T}}_{a}\delta a_{B}+\hat{R}{\left[\delta \theta \right]}_{\times }{\hat{T}}_{a}{\hat{a}}_{B}\\ \dot{\hat{\beta }} & =\hat{R}{\hat{T}}_{a}{\hat{a}}_{B} \end{array}$$

(A10) $$\begin{array}{cc} \Rightarrow \delta \dot{\beta } & =\dot{\beta }-\dot{\hat{\beta }}\\ & \approx \hat{R}{\hat{T}}_{a}\delta a_{B}+\hat{R}\delta {\hat{T}}_{a}{\hat{a}}_{B}+\hat{R}{\left[\delta \theta \right]}_{\times }{\hat{T}}_{a}{\hat{a}}_{B}\\ & =-\hat{R}{\left[{\hat{T}}_{a}{\hat{a}}_{B}\right]}_{\times }\delta \theta -\hat{R}{\hat{T}}_{a}\delta {\hat{b}}_{a}+\hat{R}\delta T_{a}{\hat{a}}_{B}-\hat{R}T_{a}n_{a}. \end{array}$$

C. The Orientation Error

We start with the following relations

(A11) $$\begin{array}{cc} \omega & =T_{\omega }\left(\omega_{m}-A_{\omega }T_{a}\left(a_{m}-b_{a}-n_{a}\right)-b_{\omega }-n_{\omega }\right)\\ & ={\hat{T}}_{\omega }{\hat{\omega }}_{B}+{\hat{T}}_{\omega }\delta \omega_{B}+\delta {\hat{T}}_{\omega }{\hat{\omega }}_{B}+\delta {\hat{T}}_{\omega }\delta \omega_{B}, \end{array}$$

where, ${\hat{\omega }}_{B}=\omega_{m}-{\hat{A}}_{\omega }{\hat{T}}_{a}{\hat{a}}_{B}-{\hat{b}}_{\omega }$, $\delta \omega_{B}=-\delta A_{\omega }{\hat{T}}_{a}{\hat{a}}_{B}+{\hat{A}}_{\omega }{\hat{T}}_{a}\delta b_{a}+\left({\hat{A}}_{\omega }+\delta A_{\omega }\right){\hat{T}}_{a}n_{a}-A_{\omega }\delta {\hat{T}}_{a}\left({\hat{a}}_{B}-\delta b_{a}-n_{a}\right)-\delta b_{\omega }-n_{\omega }$.

Substituting Equation (A11) in Equations (A3) and (A6) yields:

$$\begin{array}{c} \dot{\gamma }=\frac{1}{2}\gamma ⊗\omega ,\dot{\hat{\gamma }}=\frac{1}{2}\hat{\gamma }⊗{\hat{T}}_{\omega }{\hat{\omega }}_{B}\\ \Rightarrow \dot{\gamma }=\dot{\left(\hat{\gamma }⊗\delta \gamma \right)}=\frac{1}{2}\left(\hat{\gamma }⊗\delta \gamma \right)⊗\omega \\ \Rightarrow \dot{\hat{\gamma }}⊗\delta \gamma +\hat{\gamma }⊗\delta \dot{\gamma }=\frac{1}{2}\left(\hat{\gamma }⊗\delta \gamma \right)⊗\omega \\ \Rightarrow \frac{1}{2}\hat{\gamma }⊗{\hat{T}}_{\omega }{\hat{\omega }}_{B}⊗\delta \gamma +\hat{\gamma }⊗\delta \dot{\gamma }=\frac{1}{2}\left(\hat{\gamma }⊗\delta \gamma \right)⊗\omega \\ \Rightarrow {\left(\hat{\gamma }\right)}^{-1}\left(\frac{1}{2}\hat{\gamma }⊗{\hat{T}}_{\omega }{\hat{\omega }}_{B}⊗\delta \gamma +\hat{\gamma }⊗\delta \dot{\gamma }\right)={\left(\hat{\gamma }\right)}^{-1}\left(\frac{1}{2}\left(\hat{\gamma }⊗\delta \gamma \right)⊗\omega \right)\\ \Rightarrow \frac{1}{2}{\hat{T}}_{\omega }{\hat{\omega }}_{B}⊗\delta \gamma +\delta \dot{\gamma }=\frac{1}{2}\delta \gamma ⊗\omega \\ \Rightarrow \delta \dot{\gamma }=\frac{1}{2}\delta \gamma ⊗\omega -\frac{1}{2}{\hat{T}}_{\omega }{\hat{\omega }}_{B}⊗\delta \gamma . \end{array}$$

Remember that $\delta \gamma =\left[\begin{array}{c} 1\\ \frac{1}{2}\delta \theta \end{array}\right]$. Substitute it to the last equation above:

$$\begin{array}{cc} \left[\begin{array}{c} 1\\ \delta \dot{\theta } \end{array}\right] & =2\delta \dot{\gamma }=\delta \gamma ⊗\omega -{\hat{T}}_{\omega }{\hat{\omega }}_{B}⊗\delta \gamma \\ & =\Omega_{R}\left(\omega \right)\delta \gamma -\Omega_{L}\left({\hat{T}}_{\omega }{\hat{\omega }}_{B}\right)\delta \gamma \\ & =\left[\begin{array}{cc} 0 & -(\omega -{\hat{T}}_{\omega }{\hat{\omega }}_{B})\\ \left(\omega -{\hat{T}}_{\omega }{\hat{\omega }}_{B}\right) & -{\left[\omega +{\hat{T}}_{\omega }{\hat{\omega }}_{B}\right]}_{\times } \end{array}\right]\left[\begin{array}{c} 1\\ \frac{1}{2}\delta \theta \end{array}\right]+O\left({∥\delta \theta ∥}^{2}\right)\\ & =\left[\begin{array}{cc} 0 & -({\hat{T}}_{\omega }\delta \omega_{B}+\delta {\hat{T}}_{\omega }{\hat{\omega }}_{B})\\ \left({\hat{T}}_{\omega }\delta \omega_{B}+\delta {\hat{T}}_{\omega }{\hat{\omega }}_{B}\right) & -{\left[2{\hat{T}}_{\omega }{\hat{\omega }}_{B}+{\hat{T}}_{\omega }\delta \omega_{B}+\delta {\hat{T}}_{\omega }{\hat{\omega }}_{B}\right]}_{\times } \end{array}\right]\left[\begin{array}{c} 1\\ \frac{1}{2}\delta \theta \end{array}\right]+O\left({∥\delta \theta ∥}^{2}\right), \end{array}$$

where, $\Omega_{L}\left(\omega \right)=\left[\begin{array}{cc} 0 & -\omega^{T}\\ \omega & {\left[\omega \right]}_{\times } \end{array}\right]$, $\Omega_{R}\left(\omega \right)=\left[\begin{array}{cc} 0 & \omega^{T}\\ -\omega & -{\left[\omega \right]}_{\times } \end{array}\right]$.

Now we obtain $\delta \dot{\theta }$ as:

(A12) $$\begin{array}{cc} \delta \dot{\theta } & =\left({\hat{T}}_{\omega }\delta \omega_{B}+\delta {\hat{T}}_{\omega }{\hat{\omega }}_{B}\right)-{\left[2{\hat{T}}_{\omega }{\hat{\omega }}_{B}+{\hat{T}}_{\omega }\delta \omega_{B}+\delta {\hat{T}}_{\omega }{\hat{\omega }}_{B}\right]}_{\times }\frac{\delta \theta }{2}+O\left({∥\delta \theta ∥}^{2}\right)\\ & \approx {\hat{T}}_{\omega }\delta \omega_{B}+\delta {\hat{T}}_{\omega }{\hat{\omega }}_{B}-{\left[{\hat{T}}_{\omega }{\hat{\omega }}_{B}\right]}_{\times }\delta \theta \\ & =-{\left[{\hat{T}}_{\omega }{\hat{\omega }}_{B}\right]}_{\times }\delta \theta +\delta {\hat{T}}_{\omega }{\hat{\omega }}_{B}+{\hat{T}}_{\omega }(-\delta A_{\omega }{\hat{T}}_{a}{\hat{a}}_{B}+{\hat{A}}_{\omega }{\hat{T}}_{a}\delta b_{a}\\ & \;+\left({\hat{A}}_{\omega }+\delta A_{\omega }\right){\hat{T}}_{a}n_{a}-A_{\omega }\delta {\hat{T}}_{a}\left({\hat{a}}_{B}-\delta b_{a}-n_{a}\right)-\delta b_{\omega }-n_{\omega })\\ & \approx -{\left[{\hat{T}}_{\omega }{\hat{\omega }}_{B}\right]}_{\times }\delta \theta +{\hat{T}}_{\omega }{\hat{A}}_{\omega }{\hat{T}}_{a}\delta b_{a}-{\hat{T}}_{\omega }\delta b_{\omega }-{\hat{T}}_{\omega }{\hat{A}}_{\omega }\delta T_{a}{\hat{a}}_{B}\\ & \;+\delta T_{\omega }{\hat{\omega }}_{B}-{\hat{T}}_{\omega }\delta A_{\omega }\hat{T_{a}}{\hat{a}}_{B}+{\hat{T}}_{\omega }{\hat{A}}_{\omega }{\hat{T}}_{a}n_{a}-{\hat{T}}_{\omega }n_{\omega }. \end{array}$$

Equations (A7), (A10) and (A12) form the error-state kinematic which is same as in Equations (22)–(24).

### Appendix A.2. Mid-Point Integration for the IMU Preintegration

We perform the Mid-point numerical integration in our implementation code. This section shows the computation details.

As showed in the Figure 2, we assume that the *i*-th IMU measurement is obtained at the time instant of frame *k*, and *j*-th measurement at frame k+1. For a real sensor suite, there may be no IMU measurement at the time instant of a image frame is captured. In this case, linear interpolation method can be used to estimate a IMU measurement at the corresponding time instant. As showed in Section 3.6, the $T_{a}$, $T_{\omega }$, $A_{\omega }$ is also assumed constant in the timespan of sliding window, so we will not explicitly distinguish the $T_{a,i}$, $T_{\omega ,i}$, $A_{\omega ,i}$ and $T_{a,i+1}$, $T_{\omega ,i+1}$, $A_{\omega ,i+1}$ in the following equations.

A. Nominal-State Preintegration

Integrating Equations (19)–(21) is to get the nominal-state estimation:

(A13) $$\begin{array}{cc} {\hat{\alpha }}_{i+1}^{b_{k}} & ={\hat{\alpha }}_{i}^{b_{k}}+{\hat{\beta }}_{i+1}^{b_{k}}\delta t+\frac{1}{2}{\hat{\gamma }}_{i}^{b_{k}}{\hat{T}}_{a}\left(\frac{1}{2}\left(a_{m_{i}}+a_{m_{i+1}}\right)-{\hat{b}}_{a}\right)\delta t^{2} \end{array}$$

(A14) $$\begin{array}{cc} {\hat{\beta }}_{i+1}^{b_{k}} & ={\hat{\beta }}_{i}^{b_{k}}+{\hat{\gamma }}_{i}^{b_{k}}{\hat{T}}_{a}\left(\frac{1}{2}\left(a_{m_{i}}+a_{m_{i+1}}\right)-{\hat{b}}_{a}\right)\delta t \end{array}$$

(A15) $$\begin{array}{cc} {\hat{\gamma }}_{i+1}^{b_{k}} & ={\hat{\gamma }}_{i}^{b_{k}}⊗{\hat{T}}_{\omega }(\frac{1}{2}\left(\omega_{m_{i}}+\omega_{m_{i+1}}\right)-{\hat{A}}_{\omega }{\hat{T}}_{a}\left(\frac{1}{2}\left(a_{m_{i}}+a_{m_{i+1}}\right)-{\hat{b}}_{a_{t}}\right)-{\hat{b}}_{\omega_{t}})\delta t, \end{array}$$

where δt is the time interval between $t_{i}$ and $t_{i+1}$. At the beginning of integration, $\alpha_{b_{k+1}}^{b_{k}}$, $\beta_{b_{k+1}}^{b_{k}}$ is 0, and $\gamma_{b_{k+1}}^{b_{k}}$ is identity quaternion. The prorogation starts with i=i and repeats step by step until i+1=j.

In the following we will detail how to obtain the error-state Jacobian and covariance using the Mid-point integration.

B. Error-State of Orientation Preintegration

Define ${\bar{\omega }}_{B}=\frac{1}{2}\left({\hat{\omega }}_{B,i}+{\hat{\omega }}_{B,i+1}\right)$, ${\bar{a}}_{B}=\frac{1}{2}\left({\hat{a}}_{B,i}+{\hat{a}}_{B,i+1}\right)$. Integration Equation (A12) in discrete time:

(A16) $$\begin{array}{cc} \delta \theta_{i+1} & =\left(I-{\left[{\hat{T}}_{\omega }{\bar{\omega }}_{B}\right]}_{\times }\delta t\right)\delta \theta_{i}+{\hat{T}}_{\omega }{\hat{A}}_{\omega }{\hat{T}}_{a}\delta t\delta b_{a,i}-{\hat{T}}_{\omega }\delta t\delta b_{\omega ,i}-{\hat{T}}_{\omega }{\hat{A}}_{\omega }\delta T_{a}{\bar{a}}_{B}\delta t\\ & \;+\delta T_{\omega }{\bar{a}}_{B}\delta t-{\hat{T}}_{\omega }\delta A_{\omega }{\hat{T}}_{a}{\bar{a}}_{B}\delta t+{\hat{T}}_{\omega }{\hat{A}}_{\omega }{\hat{T}}_{a}\delta tn_{a}-{\hat{T}}_{\omega }\delta tn_{\omega }\\ & =f_{33}\delta \theta_{i}+f_{34}\delta b_{a,i}+f_{35}\delta b_{\omega ,i}+f_{36}\delta t_{a,i}+f_{37}\delta t_{\omega ,i}+f_{38}\delta a_{\omega ,i}+n_{31}n_{a}+n_{32}n_{\omega }, \end{array}$$

where,

$$\begin{array}{c} f_{33}=I-{\left[{\hat{T}}_{\omega }{\bar{\omega }}_{B}\right]}_{\times }\delta t,\;f_{34}={\hat{T}}_{\omega }{\hat{A}}_{\omega }{\hat{T}}_{a}\delta t,\;f_{35}={\hat{T}}_{\omega }\delta t,\;f_{36}=-{\hat{T}}_{\omega }{\hat{A}}_{\omega }{\left[{\bar{a}}_{B}\right]}_{T}\delta t\\ f_{37}={\left[{\bar{a}}_{B}\right]}_{T}\delta t,\;f_{38}=-{\hat{T}}_{\omega }{\left[{\hat{T}}_{a}{\bar{a}}_{B}\right]}_{T}\delta t,\;n_{31}={\hat{T}}_{\omega }{\hat{A}}_{\omega }{\hat{T}}_{a}\delta t,\;n_{32}=-{\hat{T}}_{\omega }\delta t. \end{array}$$

C. Error-State of Velocity Preintegration

Define

$$\begin{array}{cc} R_{a0} & =\frac{1}{2}{\hat{R}}_{i}{\left[{\hat{T}}_{a}a_{B,i}\right]}_{\times },R_{a1}=\frac{1}{2}{\hat{R}}_{i+1}{\left[{\hat{T}}_{a}a_{B,i+1}\right]}_{\times }\\ \bar{R} & =\frac{1}{2}\left({\hat{R}}_{i}+{\hat{R}}_{i+1}\right)\\ R_{a_{T}} & =\frac{1}{2}({\hat{R}}_{i}{\left[a_{B,i}\right]}_{T}+{\hat{R}}_{i+1}\left[a_{{B,i+1]}_{T}}\right). \end{array}$$

Integration Equation (A10) in discrete time:

(A17) $$\begin{array}{cc} \delta \beta_{i+1} & =\delta \beta_{i}-\left(R_{a0}\delta \theta_{i}+R_{a1}\delta \theta_{i+1}\right)\delta t-\bar{R}{\hat{T}}_{a}\delta t\delta {\hat{b}}_{a,i}+R_{a_{T}}\delta t\delta t_{a,i}-\bar{R}{\hat{T}}_{a}\delta tn_{a}\\ & =\delta \beta_{i}-(R_{a0}\delta \theta_{i}+R_{a1}(f_{33}\delta \theta_{i}+f_{34}\delta b_{a,i}+f_{35}\delta b_{\omega ,i}+f_{36}\delta t_{a,i}+f_{37}\delta t_{\omega ,i}+f_{38}\delta a_{\omega ,i}\\ & \;+n_{31}n_{a}+n_{32}n_{\omega }))\delta t-\bar{R}{\hat{T}}_{a}\delta t\delta {\hat{b}}_{a}+R_{a_{T}}\delta t\delta t_{a}-\bar{R}{\hat{T}}_{a}\delta tn_{a}\\ & =\delta \beta_{i}+f_{23}\delta \theta_{i}+f_{24}\delta b_{a,i}+f_{25}\delta b_{\omega ,i}+f_{26}\delta t_{a,i}+f_{27}\delta t_{\omega ,i}+f_{28}\delta a_{\omega ,i}+n_{21}n_{a}+n_{22}n_{\omega }, \end{array}$$

where,

$$\begin{array}{c} f_{23}=-\left(R_{a0}+R_{a1}f_{33}\right)\delta t,f_{24}=-\left(R_{a1}f_{33}+\bar{R}{\hat{T}}_{a}\right)\delta t,f_{25}=-R_{a1}f_{35}\delta t,\\ f_{26}=\left(-R_{a1}f_{36}+R_{a_{T}}\right)\delta t,f_{27}=-R_{a1}f_{37}\delta t,\;f_{28}=-R_{a1}f_{38}\delta t,\\ n_{21}=-\left(R_{a1}n_{21}+\bar{R}{\hat{T}}_{a}\right)\delta t,\;n_{22}=-R_{a1}n_{22}\delta t. \end{array}$$

D. Error-State of Translation Preintegration

(A18) $$\begin{array}{cc} \delta \alpha_{i+1} & =\delta \alpha_{i}+\delta \beta_{i}\delta t+\frac{1}{2}\delta {\dot{\beta }}_{i}\delta t^{2}\\ & =\delta \alpha_{i}+\delta \beta_{i}\delta t+\frac{\delta t}{2}(f_{23}\delta \theta_{i}+f_{24}\delta b_{a,i}+f_{25}\delta b_{\omega ,i}+f_{26}\delta t_{a,i}\\ & \;\;\;\;\;+f_{27}\delta t_{\omega ,i}+f_{28}\delta a_{\omega ,i}+n_{21}n_{a}+n_{22}n_{\omega }). \end{array}$$

Equations (A16)–(A18) can be represent in matrix form:

(A19) $$\begin{array}{cc} \left[\begin{array}{c} \alpha_{i+1}\\ \beta_{i+1}\\ \gamma_{i+1}\\ b_{a,i+1}\\ b_{\omega ,i+1}\\ t_{a,i+1}\\ t_{\omega ,i+1}\\ a_{\omega ,i+1} \end{array}\right] & =\underset{F_{m,i}}{\underset{︸}{\left[\begin{array}{cccccccc} I_{3} & \frac{\delta t}{2}f_{23} & I_{3}\delta t & \frac{\delta t}{2}f_{24} & \frac{\delta t}{2}f_{25} & \frac{\delta t}{2}f_{26} & \frac{\delta t}{2}f_{27} & \frac{\delta t}{2}f_{28}\\ 0 & f_{22} & I_{3} & f_{24} & f_{25} & f_{26} & f_{27} & f_{28}\\ 0 & f_{32} & 0 & f_{34} & f_{35} & f_{36} & f_{37} & f_{38}\\ 0 & 0 & 0 & I_{3} & 0 & 0 & 0 & 0\\ 0 & 0 & 0 & 0 & I_{3} & 0 & 0 & 0\\ 0 & 0 & 0 & 0 & 0 & I_{6} & 0 & 0\\ 0 & 0 & 0 & 0 & 0 & 0 & I_{9} & 0\\ 0 & 0 & 0 & 0 & 0 & 0 & 0 & I_{9} \end{array}\right]}}\left[\begin{array}{c} \alpha_{i}\\ \beta_{i}\\ \gamma_{i}\\ b_{a,i}\\ b_{\omega ,i}\\ t_{a,i}\\ t_{\omega ,i}\\ a_{\omega ,i} \end{array}\right]\\ & \;\;+\underset{G_{m,i}}{\underset{︸}{\left[\begin{array}{cccc} \frac{\delta t}{2}n_{21} & \frac{\delta t}{2}n_{22} & 0 & 0\\ n_{21} & n_{22} & 0 & 0\\ n_{31} & n_{32} & 0 & 0\\ 0 & 0 & 0 & 0\\ 0 & 0 & 0 & 0\\ 0 & 0 & 0 & 0\\ 0 & 0 & 0 & 0 \end{array}\right]}}\left[\begin{array}{c} n_{a}\\ n_{\omega }\\ n_{ba}\\ n_{b_{\omega }} \end{array}\right]. \end{array}$$

Again, the Jacobian and covariance matrix can propagate recursively:

(A20) $$J_{i+1}=F_{m,i}J_{i}$$

(A21) $$P_{i+1}=F_{m,i}P_{i}F_{m,i}^{T}+G_{m,i}QG_{m,i}^{T}.$$
